# Unusual Sequence of Events in a Case of Takotsubo Syndrome

**DOI:** 10.1155/2018/5498052

**Published:** 2018-12-13

**Authors:** C. Henriquez, R. Landau, N. Sabharwal, D. Rodriguez, V. Virparia, A. Sadiq, J. Shani

**Affiliations:** ^1^Department of Medicine, Maimonides Medical Center, Brooklyn, NY 11219, USA; ^2^Department of Cardiology, Maimonides Medical Center, Brooklyn, NY 11219, USA

## Abstract

A 73-year-old female with multiple comorbidities including coronary artery disease was admitted for an elective PCI of a lesion detected in the RCA. On the day of the planned PCI, shortly after right femoral artery cannulation, the patient developed a sudden complete heart block requiring the administration atropine and insertion of a temporary pacemaker. Concomitantly, the patient developed acute pulmonary edema, hypotension, and hypoxia requiring intubation for mechanical ventilation. Vasopressors were administered. A coronary angiogram showed patent left and right coronary arteries, unchanged when compared to the previous angiogram. An echocardiogram performed in the cardiac catheterization lab revealed global hypokinesis of the left and right ventricles, with severe LV systolic dysfunction (EF < 20%). Following an insertion of an intra-aortic balloon pump, the patient was transferred to the CICU. A repeat echocardiogram in the CICU two hours later revealed a classical echocardiographic presentation of Takotsubo syndrome, apical hypokinesis. By the next morning the patient's hemodynamic status significantly improved, the balloon pump was removed, and vasopressors were discontinued. Another echocardiogram was performed 24 hours after the event occurred and revealed a marked improvement in LV systolic function (EF 60%), with complete resolution of apical and septal wall motion abnormalities. Three days after the event, the patient was successfully discharged and asymptomatic at two-month follow-up. This case illustrates an atypical presentation of Takotsubo syndrome that was witnessed from onset to its complete resolution during the patient's hospital stay.

## 1. Introduction

First described in the 1990s, Takotsubo syndrome, also known as stress-induced cardiomyopathy and broken heart syndrome, is the acute, reversible onset of left ventricular (LV) dysfunction. Takotsubo syndrome is frequently associated with chest pain and, in some cases, can be linked to heart failure and ST segment changes that mimic acute myocardial infarction. The wall motion abnormalities are characterized by their lack of a single coronary artery distribution, and coronary angiography typically reveals no evidence of acute coronary obstruction. Despite the generally good long-term prognosis of Takotsubo syndrome, rare complications such as cardiogenic shock, LV rupture, or embolization of an LV thrombus result in a reported mortality as high as 6% [1, 2]. Additionally, malignant ventricular arrhythmia, particularly torsades de pointes associated with Takotsubo-related QT prolongation, and complete heart block can occur, though infrequently. Here, we present the case of a 73-year-old female with sudden onset and progression of Takotsubo syndrome during an elective cardiac catheterization procedure.

## 2. Case Report

**Figure 1:**
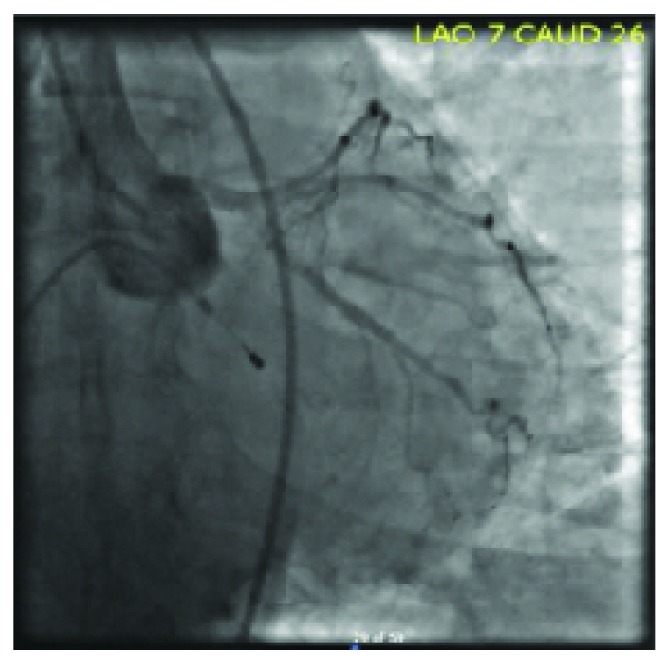
Coronary angiography showing the left coronary arteries unchanged from previous angiogram.

**Figure 2:**
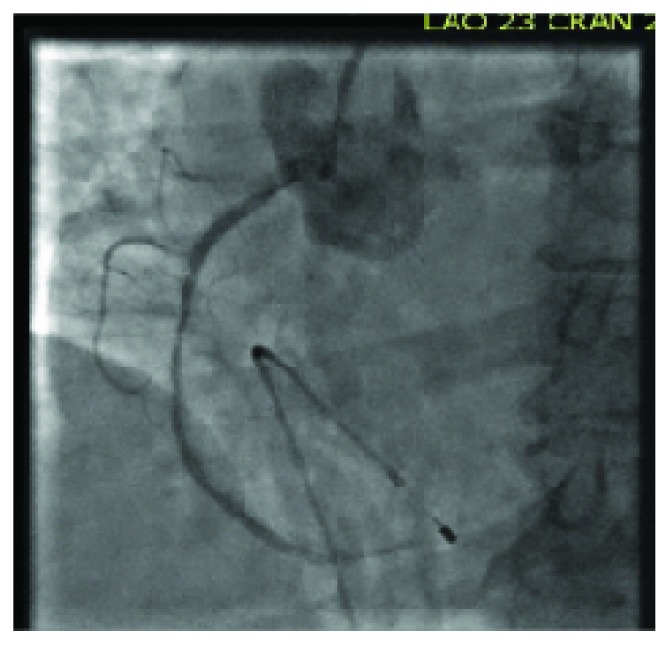
Coronary angiography showing the right coronary arteries unchanged from previous angiogram.

A 73-year-old female with a past medical history of hypertension, hyperlipidemia, insulin-dependent diabetes mellitus, and coronary artery disease was admitted for an elective percutaneous coronary intervention (PCI) of a lesion detected in the right coronary artery. The patient had old stents to the mid-right coronary artery and mid-left anterior descending artery, and a stent in the mid-left circumflex artery that was deployed three weeks prior to this admission. On the day of the planned PCI, shortly after right femoral artery cannulation, the patient developed a sudden complete heart block. Atropine was immediately administered, and a temporary pacemaker was inserted. Subsequently, the patient developed a supraventricular tachycardia (SVT) with aberrancy. IV adenosine was given, and the rhythm changed to sinus tachycardia. Concomitantly, the patient developed acute pulmonary edema and became hypotensive and hypoxic. She was intubated, started on mechanical ventilation, and vasopressors were administered to maintain her blood pressure. A coronary angiogram showed patent left and right coronary arteries, unchanged when compared to the previous angiogram (Figures 1 and 2). An echocardiogram performed in the cardiac catheterization lab revealed global hypokinesis of the left and right ventricles, with severe left ventricular systolic dysfunction (EF < 20%) (Videos 1A and 1B). Following an insertion of an intra-aortic balloon pump, the patient was transferred to the CICU. A repeat echocardiogram in the CICU two hours later revealed a classical echocardiographic presentation of Takotsubo syndrome (Videos 2A and 2B). By the next morning, the patient's hemodynamic status significantly improved, the balloon pump was removed, and vasopressors were discontinued. Another echocardiogram was performed 24 hours after the event occurred and revealed a marked improvement of the left ventricular function (EF 60%), with complete resolution of apical and septal wall motion abnormalities (Videos 3A and 3B). Three days after the event, the patient was successfully discharged. At a two-month follow-up visit, the patient was completely asymptomatic.

## 3. Discussion

In 1990, Dote and colleagues in Hiroshima, Japan, described a series of 5 patients who developed myocardial stunning due to multivessel coronary artery spasm. The end-systolic shape of the ventricle seen on the ventriculogram was described as “Takotsubo,” translated to “octopus pot” in Japanese. Hence, they named this particular condition as Takotsubo syndrome (TTS) [3].

Although Takotsubo syndrome was officially defined in the 1990s, the condition was described long before. In 1942, Cannon published a paper describing his personal observations of death by fright of the natives in Australia and New Zealand. He postulated that the sympathoadrenal system is activated when animals, including humans, are strongly aroused by emotions such as fright. This leads to a “fight or flight” response, and sometimes even to sudden death, thereby making it the most common explanation of Takotsubo syndrome pathophysiology [4].

Another theory of the pathophysiology of Takotsubo syndrome focuses on the role of catecholamines in myocardial stunning, coronary vasospasm, and microvascular endothelial dysfunction. Myocardial stunning could occur in several clinical situations, most commonly being myocardial ischemia, and can last anywhere from a few hours to multiple days [5]. Other factors contributing to myocardial stunning are oxygen free radicals and increased cytosolic calcium, which can also lead to increased propensity of cardiac arrhythmias [6–8].

Stimulus trafficking, or biased agonism, which occurs when high concentrations of epinephrine initiate a switch from G(s) protein to G(i) protein signaling in ventricular cardiomyocytes via the beta(2)-adrenoceptor could also explain the pathophysiology of Takotsubo syndrome. This switch to beta(2)-adrenoceptor-G(i) protein signaling protects against proapoptotic effects of beta(1)-adrenoceptors, which are believed to play a role in the prevention of rapid cell death in Takotsubo syndrome and help with subsequent restoration of myocardial function [9, 10]. Additionally, this switch is negatively inotropic, mostly at the apical myocardium where beta-adrenoceptor concentration is greatest, thereby explaining the apical myocardium dysfunction that occurs in Takotsubo syndrome.

Takotsubo syndrome typically presents as an array of signs and symptoms that include chest pain, EKG changes, no recognizable culprit artery on coronary angiography, and most notably, the hypokinetic or akinetic apical and midventricular myocardium and preserved or hyperkinetic basal myocardium seen on the echocardiogram or ventriculogram [11]. Our patient fortunately developed myocardial dysfunction in the cardiac catheterization lab where the team was able to act immediately and track its progression. The myocardial dysfunction began as severe global myocardial hypokinesis and progressed to the typical echocardiographic presentation of Takotsubo syndrome within 2 hours. Complete recovery of the LV systolic function surprisingly occurred within 24 hrs.

This case raises the question of whether all cases of Takotsubo syndrome start with global hypokinesis and progress to the typical echocardiographic appearance of apical hypokinesis.

To our knowledge, this is the first case report of witnessed onset and progression of Takotsubo syndrome through the different stages of the condition to its rapid complete recovery within 24 hours. A previous case report describes similar findings in a patient exposed to capecitabine chemotherapy [12]. However, our patient was not exposed to chemotherapeutic agents and the sequence of events began with global myocardial stunning which progressed to apical hypokinesis with complete recovery within 24 hours.

## 4. Conclusion

Takotsubo syndrome presents with a constellation of signs, symptoms, and electrocardiographic and echocardiographic findings as described above. Our patient had a witnessed abrupt onset of symptoms of severe myocardial dysfunction that completely resolved within 24 hours in the absence of a specific trigger which is highly unusual in cases of Takotsubo syndrome.

## Supplementary Materials

Supplementary Materials Video 1A: parasternal long-axis view revealing the global hypokinesis and severely decreased left ventricular systolic function. Video 1B: parasternal short-axis view revealing global hyperkinesis and severely decreased left ventricular systolic function. Video 2A: parasternal long-axis 2D echocardiogram performed 2 hours later revealing apical hyperkinesis with improved contractility at the base. Video 2B: apical 4 chamber 2D echocardiogram performed 2 hours later revealing apical hyperkinesis with improved contractility at the base. Video 3A: parasternal long-axis 2D echocardiogram revealing normal LV systolic function and complete resolution of apical and basal motion abnormalities. Video 3B: apical 4 chamber 2D echocardiogram revealing normal LV systolic function and complete resolution of apical and basal motion abnormalities.
