# Supplementary material for: Unusual Sequence of Events in a Case of Takotsubo Syndrome
Source: Case Rep Cardiol. 2018 Dec 13;2018:5498052. doi: 10.1155/2018/5498052 (PMC6311793; doi:10.1155/2018/5498052)

**Supplementary Video Images attached:**

Video 1A:
Parasternal Long Axis view revealing Global Hypokinesis and Severely Decreased Left Ventricu lar Systolic Function.


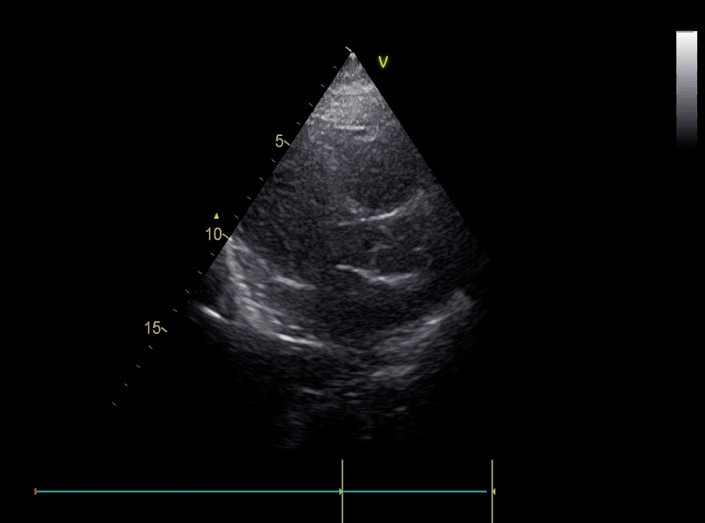


Video 1B:
Parasternal Short Axis view revealing global hyperkinesis and Severely decreased Left Ventricu- lar Systolic Function.

**
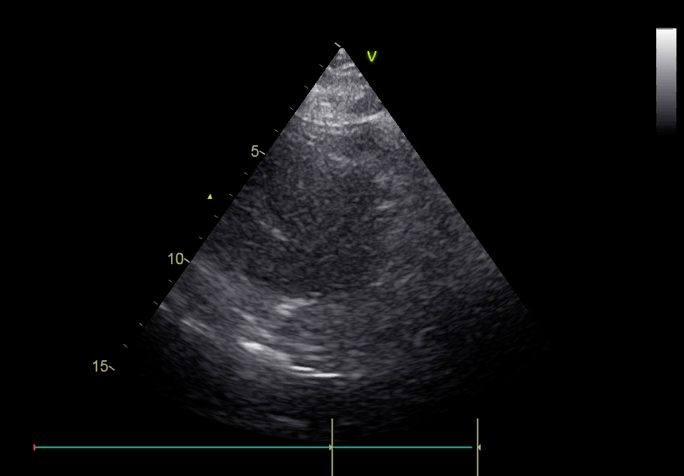
**

Video 2A:
Parasternal Long Axis 2D Echocardiogram performed 2 hours later revealing apical hyperkinesis with improved contractility at the base.


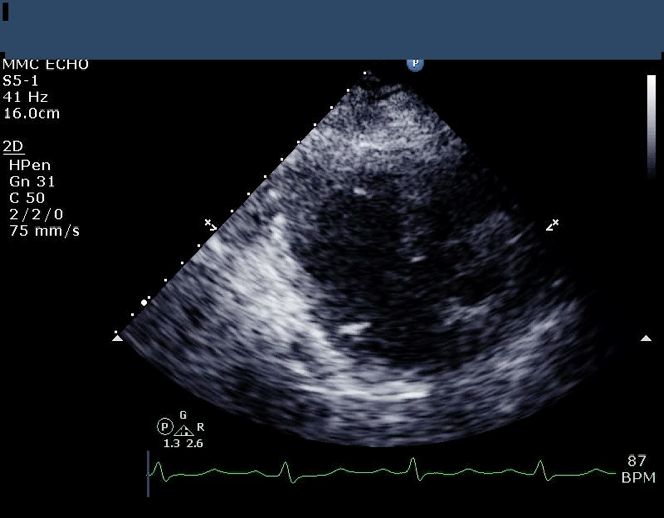


Video 2B:
Apical 4 Chamber 2D Echocardiogram performed 2 hours later revealing apical hyperkinesis with improved contractility at the base.


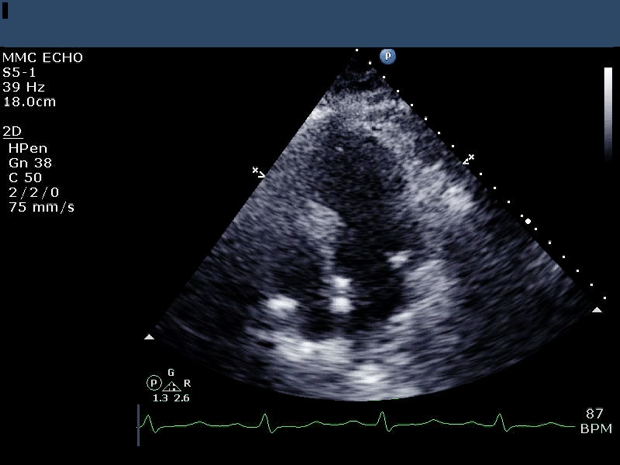


Video 3A:
Parasternal Long Axis 2D Echocardiogram revealing normal LV systolic function and complete resolution of apical and basal motion abnormalities.


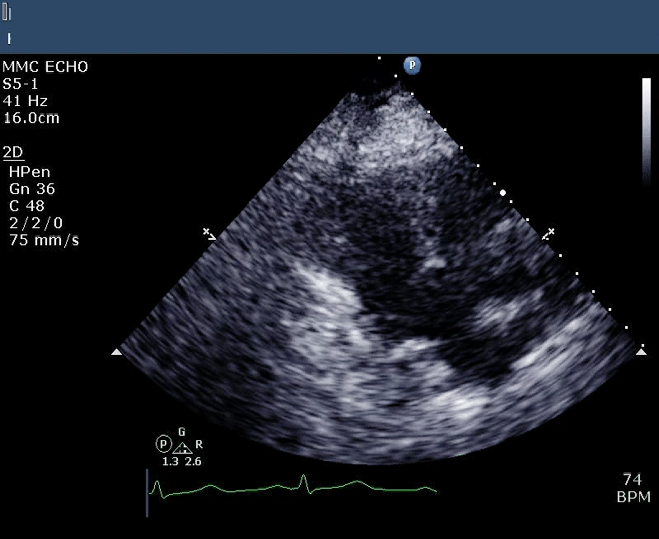


Video 3B:
Apical 4 Chamber 2D Echocardiogram revealing normal LV systolic function and complete resolution of apical and basal motion abnormalities.


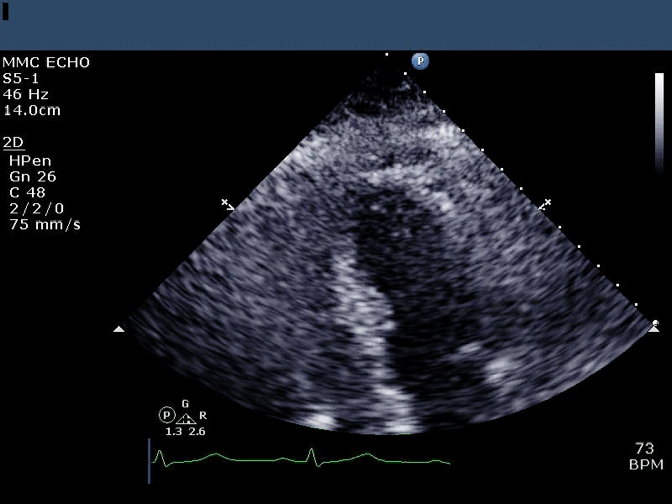

Supplement: Supplementary Materials — Video 1A: parasternal long-axis view revealing the global hypokinesis and severely decreased left ventricular systolic function. Video 1B: parasternal short-axis view revealing global hyperkinesis and severely decreased left ventricular systolic function. Video 2A: parasternal long-axis 2D echocardiogram performed 2 hours later revealing apical hyperkinesis with improved contractility at the base. Video 2B: apical 4 chamber 2D echocardiogram performed 2 hours later revealing apical hyperkinesis with improved contractility at the base. Video 3A: parasternal long-axis 2D echocardiogram revealing normal LV systolic function and complete resolution of apical and basal motion abnormalities. Video 3B: apical 4 chamber 2D echocardiogram revealing normal LV systolic function and complete resolution of apical and basal motion abnormalities. [file 5498052.f1.zip › Supplementary Video Images attached.docx]
